# A novel full‐length two‐domain *KIR2DL5A* allele isolated in Zimbabwean samples: KIR2DL5A*0010104

**DOI:** 10.1111/tan.13875

**Published:** 2020-03-30

**Authors:** Lisa van Pul, Edith Majonga, Rashida Ferrand, Sarah L. Rowland‐Jones, Louis‐Marie Yindom

**Affiliations:** ^1^ University of Oxford Nuffield Department of Medicine Oxford UK; ^2^ Department of Clinical Research London School of Hygiene and Tropical Medicine London UK; ^3^ Department of Clinical Research Biomedical Research and Training Institute Harare Zimbabwe; ^4^ Present address: Amsterdam UMC Academic Medical Center Amsterdam AZ The Netherlands

**Keywords:** *KIR2DL5*, new allele, SBT, Zimbabwe

## Abstract

The novel allele KIR2DL5A*0010104 differs from that of KIR2DL5A*0010101 with eight single intronic nucleotide changes.

1

Natural killer (NK) cells survey the immune system through various receptors expressed on their cell surface. Among these receptors are the killer‐cell immunoglobulin‐like receptors (KIR) that have major histocompatibility complex (MHC) I molecules serving as their ligands. KIR2DL5 is a two‐domain receptor variant and is presumed to have inhibitory functions based on the fact that it has immunoreceptor tyrosine‐based inhibitory motifs (ITIMs) on its cytoplasmic tail.^1^ When the receptor recognizes self‐peptide bound to MHC‐I molecules, the cytolytic function of the NK cell is inhibited preventing damage to unblemished cells.^2^ The KIR2DL5 gene exists in two variants, KIR2DL5A and KIR2DL5B, they show 99.5%‐99.7% coding sequence identity and are present at the telomeric half and the centromeric half of the KIR‐gene cluster, respectively.^3^

Described here is the identification of a novel KIR2DL5A subtype officially named KIR2DL5A*0010104 by the World Health Organization (WHO) Committee for factors of the HLA System, Subcommittee for Killer‐cell Immunoglobulin‐like Receptors. This new allele was isolated from the DNA of Zimbabwean donors using previously described high‐resolution long‐range sequencing techniques.^4^

Full‐length nucleotide sequences of KIR2DL5A*0010104 were compared with that of KIR2DL5A*0010101 and we found eight intronic single nucleotide polymorphisms four of which occurred in intron 2 as illustrated in Table 1. The nucleotide sequences of KIR2DL5A*0010104 have been deposited into GenBank with the Accession number MG004194 and IPD‐KIR database,^5^ ID: IWS40002368.

**TABLE 1:** Nucleotide sequences comparison between KIR2DL5A*0010101 and KIR2DL5A*0010104

	Chromosomal positions								
KIR allele	636	638	904	1290	1293	1334	4161	6793	
2DL5A*0010101	A	C	T	G	C	T	A	C	
2DL5A*0010104	G	T	A	A	T	G	del	A	
Location	Int 1	Int 1	Int 2	Int 2	Int 2	Int 2	Int 4	Int 5	

Abbreviation: Int, intron.

The name 2DL5A*0010104 has been officially assigned by the WHO Nomenclature Committee in October 2019. This follows the agreed policy that, subject to the conditions stated in the most recent Nomenclature Report,^6^ names will be assigned to new sequences as they are identified. Lists of such new names will be published in the following WHO Nomenclature Report.

## CONFLICT OF INTEREST

The authors have declared no conflicting interests.

2

## Data Availability

The nucleotide sequences of KIR2DL5A*0010104 have been deposited into GenBank with the Accession number MG004194 and IPD‐KIR database (5), ID: IWS40002368.
